# Genome Sequence of Pigmented Siderophore-Producing Strain Serratia marcescens SM6

**DOI:** 10.1128/MRA.00247-19

**Published:** 2019-05-02

**Authors:** Irina V. Khilyas, Kanat A. Tursunov, Tatiana V. Shirshikova, Leysan K. Kamaletdinova, Lilia E. Matrosova, Prerak T. Desai, Michael McClelland, Lydia M. Bogomolnaya

**Affiliations:** a Institute of Fundamental Medicine and Biology, Kazan Federal University, Kazan, Russia; b Laboratory of Immunochemistry and Immunobiotechnology, National Center for Biotechnology, Astana, Kazakhstan; c Department of Microbiology and Molecular Genetics, University of California Irvine, Irvine, California, USA; d Department of Microbial Pathogenesis and Immunology, College of Medicine, Texas A&M University Health Science Center, Bryan, Texas, USA; Georgia Institute of Technology

## Abstract

Here we present a draft genome sequence of laboratory strain Serratia marcescens SM6. Using the antiSMASH 5.0 prediction tool, we identiﬁed five biosynthetic gene clusters involved in secondary metabolite production (two siderophores and a biosurfactant serratamolide, a glucosamine derivative, and a thiopeptide). Whole-genome sequencing information will be useful for the detailed study of metabolites produced by Serratia marcescens.

## Abstract (precis)

Here we present a draft genome sequence of laboratory strain Serratia marcescens SM6. Using the antiSMASH 5.0 prediction tool, we identiﬁed five biosynthetic gene clusters involved in secondary metabolite production (two siderophores and a biosurfactant serratamolide, a glucosamine derivative, and a thiopeptide).

## ANNOUNCEMENT

Serratia marcescens is a Gram-negative bacterium from the family Enterobacteriaceae that can successfully adapt to different ecological niches and colonize various surfaces. It can also colonize human tissue and cause nosocomial infection of the urinary tract, respiratory tract, bloodstream, and surgical wounds (1). S. marcescens has been reported to produce an array of extracellular enzymes, including chitinases, lecithinases, lipases, proteases, and nucleases (2). It can also produce a number of secondary metabolites (3, 4). Among those, siderophores, low-molecular-weight molecules with a high affinity for iron, support the growth of bacteria when the iron supply is limited. Siderophores participate in iron chelating, transport of metals other than iron, protection against oxidative stress, and molecular signaling (5). We present the draft genome sequence and annotation of the laboratory strain S. marcescens SM6, which has been widely used in research for the past 50 years (2, 6–8) and was a gift from Michael Benedik, Texas A&M University (College Station, TX). The production of siderophores by this strain was previously reported (9); however, genetic determinants for siderophore production were not previously described.

Genomic DNA from S. marcescens SM6 was extracted from an overnight LB-grown culture with a genomic DNA isolation kit (Sigma-Aldrich). The quantity and quality of isolated DNA were determined with a NanoDrop spectrophotometer (Thermo Scientific). A paired-end sequencing library was prepared with the TruSeq DNA kit (Illumina), with an average fragment size of 600 bp, and sequenced with a 300 × 2-bp paired-end sequencing run on the MiSeq sequencing platform (Illumina). This procedure generated 2,895,172 paired-end reads. All reads were trimmed with Cutadapt version 1.5 for the Illumina universal adapter and low-quality bases with a cutoff of 20, and they were assembled with CLC Genomics Workbench version 7.5 (Qiagen) with default parameter settings, which indicated 300× coverage. Annotation was performed with the NCBI Prokaryotic Genomes Annotation Pipeline (PGAP) version 4.7. The assembly comprised 5,102,203 bp with 28 contigs in 24 scaffolds, an *N*_50_ value equal to 1,527,894, and a G+C content of 59.8%. The strain SM6 genome sequence contained 4,890 total genes, 4,736 coding sequence genes (CDS), 42 pseudogenes, and 112 RNA genes, including 82 tRNAs and 17 noncoding RNAs (ncRNAs). A search for the genetic loci required for secondary metabolite biosynthesis, using antiSMASH version 5.0 with default parameter settings (10), indicated the presence of four nonribosomal peptide synthetase (NRPS) clusters and one ribosomally synthesized and posttranslationally modified peptide cluster in the genome of S. marcescens SM6. A nucleotide sequence of gene *EG355_08745*, encoded in the NRPS for siderophore biosynthetic cluster 1, shares 70% identity with gene *cbsF* of Dickeya dadantii 3937 (11, 12). In Dickeya chrysanthemi, *cbsF* is involved in the production of the siderophore chrysobactin (12). Siderophore cluster 2 of S. marcescens SM6 contains genes similar to Serratia plymuthica V4 genes involved in the biosynthesis of the siderophore serratiochelin (4). These genes (*EG355_01020*, *EG355_01055*, *EG355_01060*, and *EG355_01045*) share 78%, 73%, 78%, and 72% nucleotide identity with *S. plymuthica* V4 genes *schF0*, *schF1*, *schF2*, and *schF3*, respectively (4). The two remaining NRPS clusters contain genes involved in the biosynthesis of a biosurfactant serratamolide (13) and a glucosamine derivative (3).

### Data availability.

This whole-genome shotgun project has been deposited at DDBJ/ENA/GenBank under the accession number SDUW00000000. The version described in this paper is SDUW01000000. Raw sequencing data have been deposited in the SRA under the accession number PRJNA505622.
